# Chronic traumatic encephalopathy neuropathologic change is associated with higher stage limbic‐predominant age‐related TDP‐43 encephalopathy

**DOI:** 10.1002/alz.084996

**Published:** 2025-01-03

**Authors:** Hailong Song, Kamar E Ameen‐Ali, Claire Kennedy‐Dietrich, Jean‐Pierre Dolle, Eddie B Lee, Douglas H Smith, William Stewart

**Affiliations:** ^1^ University of Pennsylvania, Philadelphia, PA USA; ^2^ Teesside University, Middlesbrough, North Yorkshire United Kingdom; ^3^ University of Glasgow, Glasgow, Scotland United Kingdom; ^4^ Queen Elizabeth University Hospital, Glasgow United Kingdom

## Abstract

**Background:**

Traumatic brain injury (TBI) is recognized as one major, potentially modifiable risk factor for neurodegenerative disease (NDD). Autopsy studies describe a range of neuropathologies in a proportion of individuals surviving late after TBI, most frequently the tau associated pathology, chronic traumatic encephalopathy neuropathologic change (CTE‐NC). In addition to tau, other NDD pathologies are described. Of these, deposition of abnormally phosphorylated transactive response DNA‐binding protein 43 (pTDP‐43) has been reported in association with CTE‐NC. However, to date the prevalence and distribution of pTDP‐43 in CTE‐NC and its distinction from pathology of wider NDD has not been formally assessed.

**Method:**

Patients with history of exposure to repetitive mild traumatic brain injury (rmTBI) and documented NDD (n = 30), together with age‐matched controls with no known TBI exposure, either with (n = 24) or without (n = 18) NDD, were identified within the CONNECT‐TBI archive. Whole slide digital images of standardized brain tissue sections stained for pTDP‐43 (1D3) were reviewed, and the pattern and distribution of pathology mapped.

**Result:**

Overall, prevalence of pTDP‐43 pathology was similar among rmTBI patients (40%) and their age‐matched controls with NDD (33%; p = 0.7778). However, while pTDP‐43 was typically localized in controls with NDD and in rmTBI patients without CTE‐NC (limbic‐predominant age‐related TDP‐43 encephalopathy [LATE] stage 1‐2), in patients with CTE‐NC this pathology was more often widespread and high stage (LATE stage 3; p = 0.0045).

**Conclusion:**

These results demonstrate rmTBI is associated with higher stage LATE pathology than in equivalent age matched controls and individuals with wider, non‐TBI related NDD. Further studies are required to characterize the association between TBI and TDP‐43 proteinopathy, including the contribution, if any, of this pathology to clinical sequelae of TBI related neurodegenerative disease.

